# Some Hospital Statistics: Their Use and Value

**Published:** 1916-09-09

**Authors:** 


### THE EDITOR'S LETTER-BOX.

### Some Hospital Statistics: Their Use and Value.

*To the Editor of* THE HOSPITAL.

SIR,—Your leader on hospital statistics is most interesting. Is there any chance of finding out what it costs to run a purely military hospital—*e.g.*, King George or Millbank or any of the other larger hospitals?

Then the question arises, is the War Office allowance (maximum 4s. a day, I believe) anything like adequate for the maintenance of military patients in voluntary hospitals?—Yours faithfully, HOSPITAL SECRETARY.

[The adoption of the Uniform System by order should enable the cost of a purely military hospital to be available after the war. We may remind "Hospital Secretary" that Government accounts are proverbially slow in coming to birth. On the other hand, is there any competent and experienced voluntary hospital official who is prepared to state to-day what it costs to run the purely naval and military section of a great voluntary hospital. The creation of statistics covering new ground necessitates the passing of ample time.—ED. THE HOSPITAL.]

